# Seat Belt Compression Appendicitis following Motor Vehicle Collision

**DOI:** 10.1155/2017/8245046

**Published:** 2017-03-01

**Authors:** Muhammad Faisal Khilji, Qazi Zia Ullah

**Affiliations:** Department of Emergency Medicine, Sultan Qaboos University Hospital, Muscat, Oman

## Abstract

Appendicitis and trauma both present in emergency department commonly but their presentation together in the same patient is unusual. We present a case of a middle-aged man brought by emergency medical services (EMS) to the emergency department with complaints of abdominal pain after he was involved in motor vehicle collision. He was perfectly fine before the accident. His primary survey was normal. Secondary survey revealed tenderness in right iliac fossa with seat belt mark overlying it. Computerized tomography (CT) of the abdomen and pelvis was performed which showed 8 mm thickening of appendix with minimal adjacent fat stranding. There is also subcutaneous fat stranding of anterior lower abdominal wall possibly due to bruising. Impression of posttraumatic seat belt compression appendicitis was made. Laparoscopic appendectomy was done and patient recovered uneventfully. Histopathology showed inflamed appendix, proving it to be a case of seat belt compression appendicitis.

## 1. Introduction

Traumatic appendicitis was reported in literature before. The most famous case was the death of Harry Houdini in 1926, due to rupture of appendix following repeated blows to abdomen [1]. Seat belt compression and other blunt traumas both were reported in literature as a cause of appendicitis [1, 2]. Trauma leading to edema and obliteration of appendicular lumen with subsequent inflammation of whole appendix is the proposed pathophysiology of this condition [3].

## 2. Case Report

**Figure 1:**
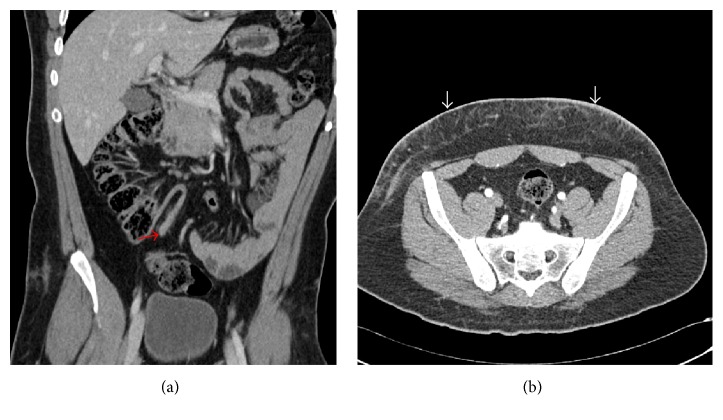
(a) CT abdomen, coronal view, showing inflamed appendix (red arrow). (b) CT abdomen, transverse view, showing subcutaneous fat stranding of lower anterior abdominal wall (white arrows).

We present a case of 43-year-old man brought by emergency medical services (EMS) to the emergency department with complaints of abdominal pain after he was involved in motor vehicle collision, two hours earlier. Patient was well before the accident. His primary survey showed intact airway, normal bilateral air entry with normal percussion, and respiratory rate of 12 per minute. No obvious injury mark was noted over chest. His blood pressure was 130/80 mm Hg, heart rate was 96 per minute, and he had normal capillary refill. The Glasgow coma scale of patient was 15/15 with blood glucose of 6 mmol/L. There was no obvious bleeding from any part of the body. Secondary survey revealed tenderness in right iliac fossa with seat belt mark overlying it. Gut sounds were audible. Ultrasound fast showed no free fluid in the abdominal cavity. Blood investigations showed white cell counts of 11 × 10/L with neutrophil predominance. Renal and liver functions were normal. Urine dipstick was negative. Computerized tomography (CT) of abdomen and pelvis was performed which showed 8 mm thickening of appendix with minimal adjacent fat stranding (Figure 1(a)). There is also subcutaneous fat stranding of anterior lower abdominal wall possibly due to bruising (Figure 1(b)). Impression of posttraumatic seat belt compression appendicitis was made. Laparoscopic appendectomy was done. Appendix was in pelvic position and 10 cm in length. Patient recovered uneventfully. Histopathology showed inflamed appendix.

## 3. Discussion

It is very rare for the appendix to be affected by trauma due to its small size and free mobility in the abdominal cavity. Traumatic appendicitis was reported in literature before. A fall on bicycle handle bar, fall from ladder with abdominal injury, and motor vehicle accidents were all reported in literature as cause of traumatic appendicitis [2, 4]. The overall incidence of people undergoing appendectomy for acute appendicitis is approximately 7% with its peak between second and fourth decade of life [5, 6]. The lifetime chance of having appendectomy is 12% in males and 25% in females [5, 6]. Obstruction of appendix lumen is believed to be the major cause of appendicitis. Other causes include mucosal or submucosal inflammation, leukemia, and endometriosis [6, 7]. Mucosal or submucosal inflammation secondary to increased cecal and appendicular pressure is believed to be the major cause of traumatic appendicitis as proposed by Hennington et al. [2, 8]. The relationship between trauma and appendicitis is not clearly understood but cases were reported pointing trauma as a case of appendicitis [1, 2]. Probability of appendix being affected by trauma depends on its position also. As in our case appendix was in pelvic position and 10 cm in length. Retrocaecal appendix is extremely unlikely to be injured in isolation whereas appendix positioned in inguinal hernia (Amyand's hernia) is comparatively more vulnerable to traumatic injury [9]. Possible mechanism of traumatic appendicitis is blunt trauma increasing intraluminal pressure of intestine, colon, and appendix subsequently causing edema of appendicular orifice and wall leading to inflammation of appendix [10]. Lack of abdominal pain preceding trauma, short duration blunt abdominal force, and worsening signs and symptoms leading to clinical presentation of appendicitis confirmed and relieved by surgery are three diagnostic criteria of traumatic appendicitis proposed by Ramsook [11]. Right ovarian pathologies in females and rectus sheath hematoma in both males and females are the main differentials. Diagnosis is made by clinical examination. Ultrasound abdomen and CT abdomen are helpful tools in confirming the diagnosis. Laparoscopic appendectomy is required in most of the cases.

## 4. Conclusion

Appendicitis should be suspected in trauma patients presenting with right iliac fossa pain especially with seat belt mark over the same area.
